# Assessment of Knowledge and Attitude of Anaesthetists in Utilizing Telehealth-Based Pre-anaesthesia Evaluation

**DOI:** 10.7759/cureus.51663

**Published:** 2024-01-04

**Authors:** Faris AlDobekhi

**Affiliations:** 1 Anesthesia, Majmaah University, Majmaah, SAU

**Keywords:** evaluation, pre-anaesthetic, telemedicine, telehealth, anaesthesiologist

## Abstract

Background: This study aims to assess anaesthesiologists' understanding and attitudes toward utilizing telehealth for pre-anaesthesia evaluations (PAEs) in instances where a scheduled surgery is deferred to the procedure day due to hospital or patient-related reasons.

Methodology: This observational cross-sectional study involved anaesthesiologists with over six months of hospital experience, opting to participate voluntarily. Non-probability sampling was employed for participant selection. The study's objectives were communicated, and consent was obtained. Data were recorded in Microsoft Excel and analyzed using STATA 12.0.

Results: Of the 237 participating anaesthetists, 155 were aged 21 to 40. Notably, 88.6% (n=210) expressed interest in advanced telemedicine learning, and 77.6% (n=184) were keen on its implementation. Common sources of information included tele-diagnosis (n=194), tele-education, counselling (n=147), and tele-surveillance, with additional input from telesurgery, tele-triage, tele-monitoring, and teleradiology.

Conclusion: The study highlights anaesthetists' strong enthusiasm for adopting advanced telemedicine and teleconferencing. Predominant information sources included tele-diagnosis, tele-education, tele-counselling, and tele-surveillance. The majority endorsed the potential of telemedicine to aid patients, expressing comfort in using it for pre-anaesthesia examinations.

## Introduction

When a surgeon plans an operation for a specific date, the anaesthesia department approves the procedure, and the required medical supplies and equipment are available and ready, but the surgery is postponed to the day of the procedure by either the hospital or the patient [1]. A metric used to assess the management system's efficiency and capacity to deliver high-quality patient care is the rate of surgical cancellations. There are many reasons why an operation could need to be delayed, including poor scheduling, equipment shortages, insufficient patient optimization, patient resistance, or no-shows. The most common cause of cancellations is a lack of time or resources [1].

It's common for cancellations to occur without warning. Many institutions investigate the frequency and underlying causes of cancellations before putting a strategy in place to reduce them. It is unclear if cancellations resulting from inpatient or outpatient circumstances have the same causes. According to research by Xue et al., inadequate pre-operative planning was the key factor in the study groups' same-day cancellations [2]. "High international normalized ratio (INR)" and "not nil per os (NPO)" were mentioned as the reasons the case could not proceed. Due to these problems, the patients would need further testing prior to surgery, which would cause delays and cancellations. If a healthcare provider had the opportunity to visit with the patient beforehand and go over instructions concerning things like which medications should be taken before surgery and the need for NPO adherence, these problems might have been avoided. A change in a patient's medical condition was shown to be the second most common cause for cancellation [3]. In research by Fayed et al., four patients' no-shows accounted for 27% of cancellations, whereas poorly optimized patients accounted for 24.1%, operating room (OR) unavailability for 19.3%, and patient rejection for 8.8%. Except for a lack of OR capacity, the majority of these occurrences are related to poor patient compliance and education [4].

Examining anaesthesiologists' knowledge and attitudes toward telehealth in the context of surgical cancellations is crucial for addressing factors contributing to delays. This assessment informs strategies to streamline pre-operative processes, optimize resource allocation, and identify and mitigate barriers to telehealth adoption that may impact cancellations. It contributes to improving overall surgical efficiency, minimizing disruptions, and ensuring that telehealth practices align with the perspectives and expertise of healthcare professionals. Ultimately, understanding anaesthesiologists' attitudes supports targeted interventions to reduce cancellations and enhance the integration of telehealth technologies in the surgical workflow. This study intends to evaluate the pre-anaesthesia evaluation (PAE) knowledge and attitude of anaesthesiologists via telehealth.

## Materials and methods

This observational cross-sectional study was conducted online and the questionnaire was distributed among anaesthesiologists working in different hospitals of the Riyadh region from the period of March 2023 to June 2023. After the ethical approval was obtained from the institutional review board (IRB) committee of Majmaah University with IRB number MUREC-Mar.1/ COM-2023/10-3, the questionnaire was distributed among different social media platforms, such as WhatsApp, Facebook, and Twitter, and was meant for anaesthesiologists of the central region of Saudi Arabia. Anaesthesiologists who were posted in the hospital for more than six months of period and willing to participate were included in the study.

Study tools

On the G-form, a pre-tested, semi-structured questionnaire was developed. It was divided into three sections. The consent section was the first and included a brief description of the study's goal. The participants' brief demographic information was included in the second section, along with questions on their knowledge and awareness of telemedicine in PAE. The third section had questions based on the participant's knowledge and awareness of the type of anaesthesia provider and years of experience. Their opinions towards telemedicine and their propensity to adopt it into clinical practice were assessed using a 5-point Likert scale in the questions. The tool was validated by a team of experts (anaesthesia, public health, biostatistician and a basic sciences expert).

Study procedure

The study's purpose was explained to all anaesthesiologists, and their agreement was obtained digitally. The study only included those who gave their consent and expressed a willingness to participate. They were given access to the Google Form. Once the individuals had finished filling out the form, they submitted it themselves. Exclusion criteria include anaesthesiologists who had been posted for less than six months to ensure that participants had sufficient experience in their roles. Participants who provided incomplete or irrelevant responses in the questionnaire were excluded from the data analysis to ensure the quality and validity of the collected data. A 10% non-response rate and attrition was observed among the participants.

Operational definitions

Telehealth 

It is defined as “the use of electronic information and telecommunications technologies to support long-distance clinical health care, patient and professional health-related education, public health and health administration. Technologies include videoconferencing, the internet, store-and-forward imaging, streaming media, and terrestrial and wireless communications. Telehealth is occasionally referred to as telemedicine. Although the terms are similar, they are not the same. Telehealth refers to a broader scope of health services than telemedicine does" [5].

Pre-anaesthesia Evaluation

PAE is defined as “the process of clinical assessment that precedes the delivery of anaesthesia care for surgery and for nonsurgical procedures" [6]. Several aspects are involved in the PAE, including the patient’s medical records (PMR), the interview, the physical exam, and results from diagnostic tests. The assessments made during this evaluation can be used to develop a plan of care for the patient, organize resources for perioperative care, and educate the patient.

Data analysis

The data was imported into Microsoft Excel, and STATA 12.0 was used for the analysis. Depending on whether the data were normal (as determined by a visual examination of the Q-Q plot), the quantitative variables were summarised as mean (standard deviation) or median (IQR). The frequency and percentage represent a summary of the category variables. chi-square tests was used for assessing associations between variables and descriptive statistics for summarizing participant characteristics and attitudes.

## Results

A total of 237 anaesthetists participated in the study; most of them were 21-40 years (n=155). Approximately 35% of each of them were either posted as consultants (n=84, 35.4%) or residents (n=87, 36.7%). About half of them (n=97, 49%) had experience of one to five years, while one-fourth of them had experience of more than 15 years (Table 1).

**Table 1:** Background characteristics of the study participants

Background characteristics	Count (n)	Percentage (%)
Age category		
21-40	155	68.3 %
41-60	65	28.6 %
More than 60	7	3.1 %
Highest education qualification		
Consultant	84	35.4 %
General practitioner	33	13.9 %
Professor	8	3.4 %
Resident	76	32.1 %
Specialist	36	15.2 %
Profession		
Anaesthesia consultant	85	35.9 %
Anaesthesia professor	10	4.2 %
Anaesthesia resident	87	36.7 %
Anaesthesia services	16	6.8 %
Anaesthesia specialist	39	16.5 %
Years of experience categories		
01-05 years	97	49.0 %
06-10 years	25	12.6 %
11-15 years	27	13.6 %
>15 years	49	24.7 %

It was observed that three-fourths of them (n=184, 77.6%) were interested in implementing advanced telemedicine, and four out of five of them were interested in learning about it (n=210, 88.6%). There were 161 participants (67.9%) who had attended a teleconference. The statement that "Telemedicine used in PAE has a role in reducing morbidity in case of head and neck surgery” was supported by 45.1% of them (Table 2).

**Table 2:** Distribution of study participants on the basis of their interest in telemedicine PAE: Pre-anaesthesia evaluation

Interest-based questions	n	%
Interested in implementing advanced telemedicine technologies	184	77.6%
Interested in learning about telemedicine	210	88.6%
Ever attended any teleconference	161	67.9%
Telemedicine used in PAE has a role in reducing morbidity in case of head and neck surgery	107	45.1%

**Figure 1:**
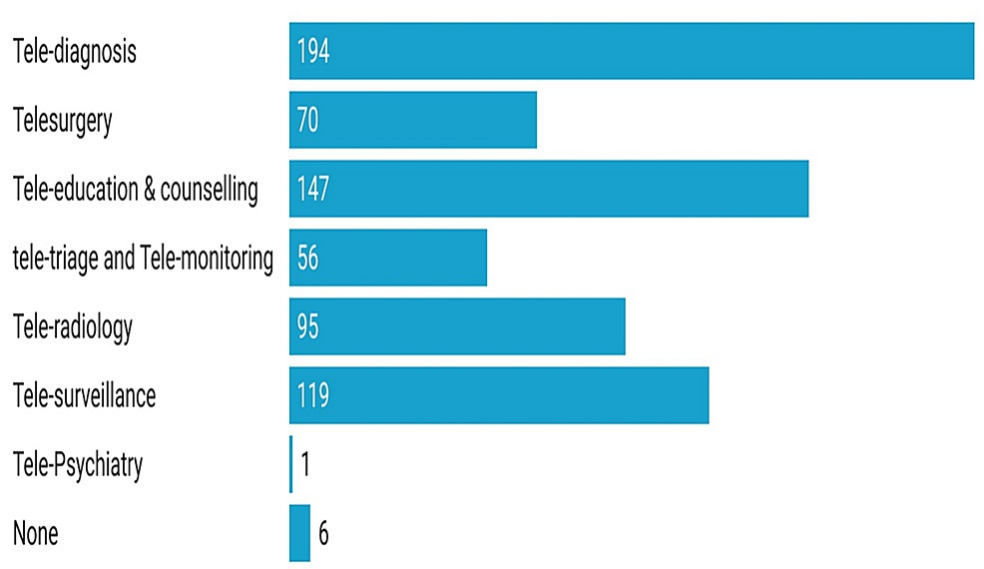
Source of information about telemedicine as stated by the study participants

The most common source of information for the participants was tele-diagnosis (n=194), tele-education and counselling (n=147), and tele-surveillance. Other sources were telesurgery, tele-triage and tele- monitoring and teleradiology (Figure 1).

**Figure 2:**
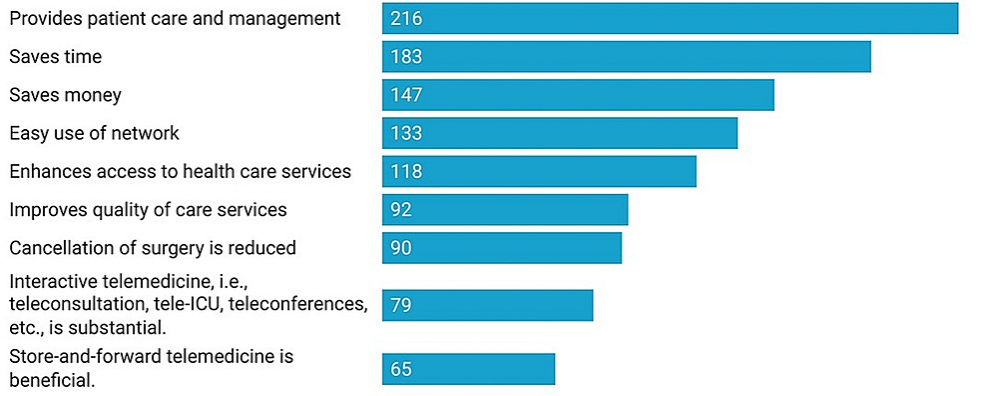
Benefits of telemedicine as cited by the anaesthetists

Most of the participants stated that telemedicine is beneficial in terms of providing patient care and management (n= 216), saving time (n= 183) and money (n= 147). Out of 237, 118 anaesthetists claimed that it enhances the accessibility of health care and usage of the network (n=133). Similarly, better quality of care (n=92) and reduction in cancellation of surgery (n=90) were claimed. Interactive sessions (n=79), and the facility of storing and forwarding the data (n=65) were mentioned by a few anaesthetists (Figure 2).

**Figure 3:**
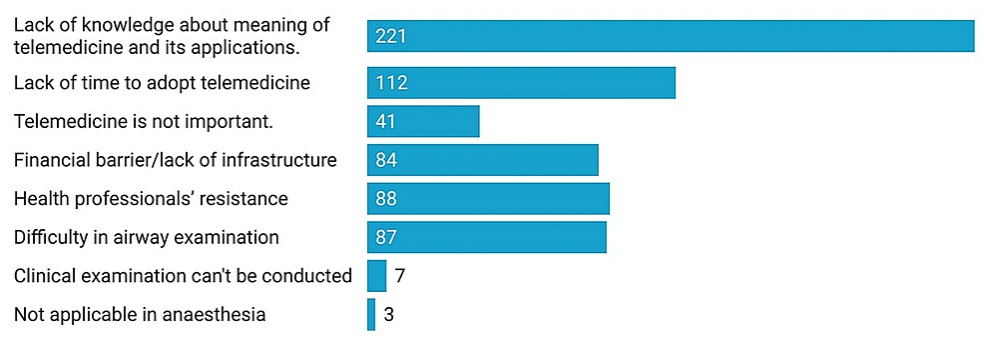
Barriers to implementing telemedicine at the anaesthetist’s level

The anaesthetists felt that the major challenge in the implementation of telemedicine is the lack of knowledge and its application (n=221). Out of 237, 88 cited resistance of health professionals as a main barrier to implementing telemedicine. Apart from these, financial barriers were pointed out by 84 of them. There were a few participants who felt that there was no role of telemedicine in anaesthesia. (n=41) and it is not applicable (n=3). Few participants feared that with telemedicine, clinical examination could not be performed (Figure 3).

**Figure 4:**
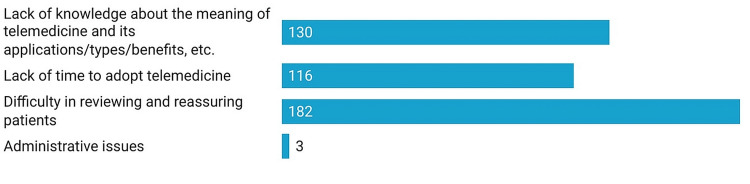
Barriers to implementing telemedicine at the hospital level

The most common barrier at the hospital level in the implementation of telemedicine cited was difficulty in reviewing the patients and reassuring them (n=182). Other reasons were a lack of knowledge about telemedicine (n=130) and time to adapt to new technology (n=116) (Figure 4).

The anaesthetists were enquired about their attitude regarding telemedicine using a set of attitude-based questions. It was observed that, overall, less than half of the participants strongly agreed with the statements. Two-fifths of them felt comfortable using telehealth to perform the PAE (n=96, 40.5%) and supported the fact that it could benefit patients (n=93, 39.2%). An approximately equal proportion of them felt it would be an effective way to perform PAE (n=76, 32.1%), and they could effectively address concerns and questions my patients may have through the telehealth-assisted PAE (n=73, 30.8%). Only one-fourth of them felt that their patients would be satisfied with telemedicine sessions for PAE (n=60, 25.3%). Only one out of five affirmed that they would encourage the use of telehealth-assisted PAEs at their institution (n=53, 22.4%) and that it would reduce the cost of day-of-surgery cancellations (n=48, 20.3%). Still, a lesser number of anaesthetists were of the opinion that it would not interfere with the privacy of the patients (n=45, 19%) and airway examination (n=32, 13.5%) (Table 3).

**Table 3:** Distribution of participants with positive attitudes towards telemedicine PAE: Pre-anaesthesia evaluation

Attitude-based questions regarding telemedicine	Participants who strongly agree with the statement	
	n	%
I would feel comfortable using telehealth to perform the PAE	96	40.5
My patients could benefit from a PAE performed through telehealth	93	39.2
Utilizing telehealth is an effective way to perform the PAE	76	32.1
I can effectively address concerns and questions my patients may have through the telehealth-assisted PAE	73	30.8
My patients would be satisfied if I performed the PAE using telehealth	60	25.3
I would encourage the use of telehealth-assisted PAEs at my institution when indicated	53	22.4
Telehealth-assisted PAEs can help reduce day-of-surgery cancellations in my institution	48	20.3
The privacy of my patients is maintained when telehealth is utilized for the PAE	45	19.0
Telehealth-assisted PAEs can reduce costs to both my institution and my patients	44	18.6
An airway assessment can be effectively performed through telehealth with video 3.43	32	13.5

There were only 26.2% of the participants who claimed to be currently using the telemedicine platform. There were 46.8% (n=111) of them who were using this for PAE. Further, 86 of them were using it since the pre-COVID era and 146 of them (61.8%) intended to use it even after COVID. A total of 14.7% of the participants were using the telemedicine platform for two-three years, while 73% of them did not respond to this. Apart from using the telemedicine platform for PAE, a few anaesthetists were used for pre-operative (n=94, 39.7%), post-operative (n=17, 7.2%) and pain management (n=33, 13.9%) purposes. Two-fifths of them (n=94, 39.4%) claimed that the average time for a single telemedicine health visit was 21-25 mins, while one-fifth of them (n=49, 20.7%) felt it took 5-10 mins. The participants invariably used different platforms for telemedicine sessions, so we received 315 responses. The most common among them were the hospital telemedicine platform (n=158, 66.7%) and the Zoom platform (n=106, 44.7%). Other less commonly used platforms were Webex, Skype, face time, ZocDoc, etc. (Table 4).

**Table 4:** Distribution of study participants based on the practice of telemedicine PAE: Pre-anaesthesia evaluation; PMR: Patient’s medical records

Practice-based questions about telemedicine	n	%
Currently using Telemedicine	62	26.2%
Using telemedicine platform for PAE	111	46.8%
Using telemedicine platforms since the pre-COVID era	85	35.9%
Intend to use telemedicine in your practice after COVID-19	146	61.6%
Years of experience using telemedicine		
≤1 year	22	9.3%
2-3 years	35	14.8%
4-5 years	3	1.3%
>5 years	2	0.8%
None	175	73.8%
Other reasons to use telemedicine platforms apart from PAE		
Pre-operative	94	39.7%
Post-operative	17	7.2%
Pain Clinic	33	13.9%
Other (Diagnostics, High-risk patient management, Conference, PMR)	6	2.5%
Not using	87	36.7%
Average time taken for a single telemedicine health visit		
1-5 mins	18	7.6%
5-10 mins	49	20.7%
11-15 mins	16	6.8%
16-20 mins	5	2.1%
21-25 mins	94	39.7%
>30 mins	7	3.0%
No answer	48	20.3%
Platforms used for Telemedicine sessions		
Zoom	106	44.7%
Webex	14	5.9%
Hospital Telemedicine platform	158	66.7%
Skype	16	6.8%
Face Time	15	6.3%
Zocdoc	5	2.1%
Doximity	1	0.4%

Table 5 depicts the factors which are associated with the practice of telemedicine for PAE.

**Table 5:** Association between the practice of telemedicine for PAE and various factors PAE: Pre-anaesthesia evaluation

Features	Currently using telemedicine for PAE			P-value (chi-square test )
Age categories	No	Yes	Total	
21-40	94	69	163	0.003
	57.7 %	42.3 %	100.0 %	
41-60	24	41	65	
	36.9 %	63.1 %	100.0 %	
>60	6	1	7	
	85.7 %	14.3 %	100.0 %	
Interested in implementing telemedicine				
No	41	10	51	<0.001
	80.4 %	19.6 %	100.0 %	
Yes	83	101	184	
	45.1 %	54.9 %	100.0 %	
Interested to learn about telemedicine				
No	21	4	25	0.001
	84.0 %	16.0 %	100.0 %	
Yes	103	106	209	
	49.3 %	50.7 %	100.0 %	
Ever attended a teleconference				
No	46	28	74	0.05
	62.2 %	37.8 %	100.0 %	
Yes	78	83	161	
	48.4 %	51.6 %	100.0 %	

It was observed that those aged 41-60 years were more likely (63.1%) to practise telemedicine compared to those between 21-40 years (42.3%) and more than 60 years (14.3%) (p=0.003). Those who were interested in learning (50.7%), implementing (54.9%) and having attended a teleconference (51.6%) were significantly associated with the usage of telemedicine platform for PAE.

**Figure 5:**
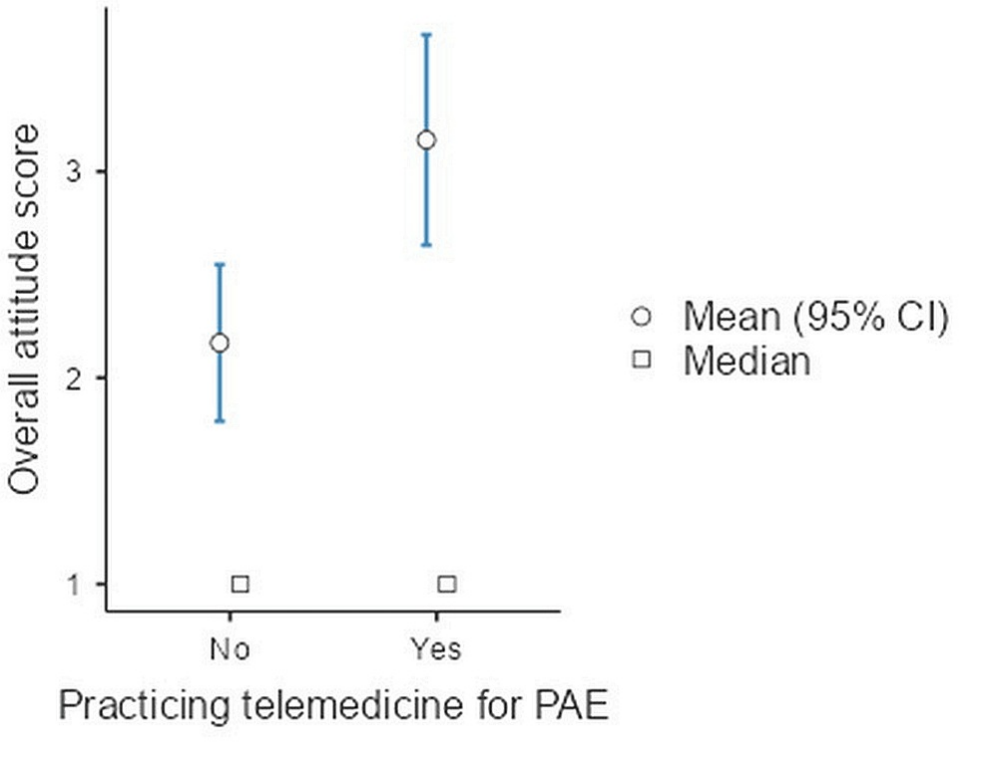
Comparison of mean attitude scores between telemedicine-practising and non-practising anaesthetists PAE: Pre-anaesthesia evaluation Total range/scale of the attitude score: 1 to 5

It is evident from Figure 5 that those who were practising telemedicine (3.15 (+/-2.74) significantly had better overall attitude scores than those who were not (2.17 (+/- 2.16)) (p=0.003)

## Discussion

In the suggested study, it was found that those between the ages of 41 and 60 were more likely (63.1%) than those between the ages of 21 and 40 (42.3%) and those above 60 (14.3%) to practise telemedicine (p 0.003). The use of a telemedicine platform for PAE was substantially associated with those who were interested in learning (50.7%), implementing (54.9%), and having attended a teleconference (51.6%) whereas in a mixed-methods study [5], Researchers sought to report health system and patient experiences with the implementation of a telehealth scheduled video visit programme across a primary care health system in a study that included a descriptive section that looked at the implementation of a telehealth scheduled visit programme at a large urban academic-affiliated health system. A survey of the study participants' patients was also conducted as a part of the study. The researchers put into practice the use of a program called Jeff Connect. The same procedures used to schedule in-person appointments apply to scheduling telemedicine appointments. Patients would use a laptop or desktop with a microphone and webcam to check in to their account on the day of their booked session. Then, the meeting would go on as usual. A total of 746 providers were trained to use the telehealth visits.

Less than half of the participants in the suggested study were found to strongly agree with the claims overall. Two-fifths of them (n=96, 40.5%) were at ease conducting the pre-anaesthesia examination through telehealth and agreed that it might be advantageous for patients (n=93, 39.2%). A roughly equal number of them believed it would be an efficient approach to do the pre-anaesthesia examination (n= 76, 32.1%) and that they could answer any queries and concerns my patients could have through the telehealth-assisted PAE (n= 73, 30.8%). Only one-fourth of them (n=60, 25.3%) thought that telemedicine sessions for PAE would be satisfactory for their patients whereas in telemedicine was used to conduct 3018 outpatient consultations during an 18-month period, and 764 of these patients completed surveys following their appointments. Most of these patients (84.8%) hadn't previously used telehealth. The majority (86%) firmly concurred that using the app made accessing care easier. Additionally, 91% of patients said they had enough time with the clinician, 82.7% said they thought they received the same level of care as in-person visits, and 5 patients said they did not appreciate communicating on video [5].

A finding in the current study indicated that 46.8% (n=111) of them were using this for PAE. Furthermore, 146 of them (61.8%) planned to continue using it even after COVID, while 86 of them had been doing so since before COVID. 14.7% of the participants in total reported utilizing the telemedicine platform for two to three years, while 73% of them did not reply. A few anaesthetists also used the telemedicine platform for pre-operative (n=94, 39.7%), post-operative (n=17, 17.2%), and pain management (n=33,13.9%) purposes, in addition to PAE. One-fifth of them (n=49, 20.7%) said it took 5-10 minutes, while two-fifths (n=94, 39.4%) said the average length of a single telemedicine health visit was 21-25 minutes. The participants invariably used different platforms for telemedicine sessions, so we received 315 responses, whereas in a retrospective study, the efficiency of telemedicine in terms of time, distance, and cost savings at a cancer centre was investigated by researchers. On June 29, 2020, the institution began offering telehealth-assisted PAE to some patients. The study compared the initial 120 patients who experienced telemedicine-based pre-anaesthetic examination to the initial 120 patients who underwent in-person PAE prior to the introduction of telehealth. Chart analysis was used to gather all the data [6]. Day-of-surgery cancellations were 1.67% in the telemedicine group versus 0% in the in-person cohort; the difference in cancellations between the two groups was not statistically significant (P value=0.4979); the majority of patients in the review fell into the ASA 2 or ASA 3 physical status categories (American Society of Anesthesiologists Physical Status Classifications) in both the telemedicine and in person visit categories [6].

Furthermore, the two cancellations that happened in the telehealth group were brought on by events beyond the control of the anaesthesia provider. Each patient from the telemedicine group saved an average of 121 minutes of time and 80 miles of distance on their round-trip travel. The researchers calculated that each patient would save an average of $46 on petrol [6].

In the suggested study, only one out of five affirmed that they would encourage the use of telehealth-assisted PAEs at their institution (n=53, 22.4%) and that it would reduce the cost of day-of-surgery cancellations (n=48, 20.3%). In a descriptive study, the group that utilized telemedicine experienced a day-of-surgery case cancellation rate of 2.95%, whereas the in-person cohort had a slightly higher rate of 3.23%. In morning and afternoon traffic circumstances, respectively, telemedicine patients avoided a median round-trip driving distance of 63 miles (Q1 24; Q3 119) and a median time saved of 137 (Q1 95; Q3 195) and 130 (Q1 91; Q3 237) minutes [7]. Future pre-anaesthetic consultations may benefit from telemedicine just as much as in-person consultations. During a pandemic, in-person consultations will cost more and need more time and resources. In the present study, 86 participants were using it since the pre-COVID era and 146 of them (61.8%) intended to use it even after COVID [8-10].

The field of telemedicine in anaesthesia will also act as a new platform for the creation of models that can promote better recovery after operations, whereas in the present study, apart from using the telemedicine platform for PAE, a few anaesthetists were used for pre-operative (n=94, 39.7%), post-operative (n=17, 7.2%) and pain management (n=33, 13.9%) purposes [11-15].

This study has some limitations such as anaesthetists who were more interested or actively involved in telemedicine might have been more likely to participate, potentially skewing the findings towards a more positive attitude towards telemedicine. The study's results may not be fully representative of all anaesthesiologists, limiting the generalizability of the findings to a broader population of healthcare professionals. Self-reporting can introduce response bias, as participants may provide answers they perceive as socially desirable or may not accurately recall their experiences or attitudes. This may impact the accuracy of the reported attitudes and behaviors related to telemedicine. Longitudinal studies that follow participants over an extended period could provide deeper insights into the evolving perceptions and behaviors of anaesthesiologists regarding telemedicine. A more diverse sample could have provided a broader perspective on telemedicine adoption. These limitations underscore the need for caution when interpreting the study's findings and highlight areas for further research and exploration in the field of telemedicine adoption among anaesthesiologists.

Our study sheds light on the practical implications of telemedicine in PAE. The finding that three-fourths of participants express interest in advanced telemedicine suggests a positive attitude toward embracing technology in healthcare. The reported benefits, such as improved patient care, time and cost savings, and enhanced accessibility, underscore the potential advantages of telemedicine in streamlining healthcare delivery. The study's focus on reducing surgery cancellations aligns with the broader goal of optimizing healthcare resources.

The results suggest potential areas for further research, particularly in addressing the barriers identified. Exploring strategies to overcome the stated challenges, such as improving knowledge and addressing resistance among health professionals, could be valuable. Additionally, investigating the impact of telemedicine on patient satisfaction, privacy concerns, and clinical examination feasibility may provide insights for refining telehealth practices in anaesthesia.

The study acknowledges potential bias, specifically mentioning the overrepresentation of anaesthesiologists interested in telemedicine. This could impact the generalizability of findings and calls for caution in applying the results universally. The study should be interpreted in the context of the sample's predisposition towards telehealth, and efforts should be made to diversify the participant pool for a more comprehensive understanding.

## Conclusions

The study revealed that anaesthetists were enthusiastic about implementing cutting-edge telemedicine and teleconferences. The participants' most popular information sources were tele-diagnosis, tele-education, tele-counselling, and tele-surveillance. The majority of them approved the idea that telemedicine may help patients and felt comfortable using it to do the pre-anaesthesia examination.
